# The mature EV71 virion induced a broadly cross-neutralizing VP1 antibody against subtypes of the EV71 virus

**DOI:** 10.1371/journal.pone.0210553

**Published:** 2019-01-16

**Authors:** Chia-Ying Wu, Shu-Ling Yu, Yung-Tsung Chen, Yi-Hsuan Chen, Pei-Wen Hsiao, Yen-Hung Chow, Juine-Ruey Chen

**Affiliations:** 1 Adimmune Corporation, Taichung, Taiwan; 2 Institute of Infectious Disease and Vaccinology, National Health Research Institutes, Zhunan, Miaoli County, Taiwan; 3 Agricultural Biotechnology Research Center, Academia Sinica, Taipei, Taiwan; 4 Graduate Institute of Biomedical Sciences, China Medical University, Taichung, Taiwan; University of Massachusetts Medical School, UNITED STATES

## Abstract

Enterovirus 71 (EV71) has emerged as a neurological virus causing life-threatening diseases in young children and infants. Although EV71 vaccines in development have presented promising results in several clinical trials, the identified key antigen for improving the broad protective efficacy of EV71 vaccines has not been well investigated. In this report, we show that different multiplicities of infection (MOIs) of the B4(E59) virus significantly affect EV71 vaccine production in a serum-free microcarrier bioreactor system. The antigens produced from high MOIs of 10^−1^ and 10^−2^ exhibited higher yield and more infectious full particle (FP) contents in the EV71 vaccines than those produced with low MOIs of 10^−4^ and 10^−6^, leading to better cross-neutralizing efficacy. The C4(E36) neutralization results showed that only antisera raised from EV71 FPs provided substantial neutralizing titers against C4(E36), whereas empty particles (EPs) of EV71 conferred no efficacy. Competitive ELISA showed that anti-FP mainly binds to FPs and that 20% of antibodies bind to EPs, whereas most anti-EP binds EPs, with only 10% antibodies binding to FPs. VP1-adsorbed anti-FP lost most of the virus neutralization efficiency, suggesting that the VP1 subunit of FP is the major immunogenic antigen determining the ability of the EV71 vaccine to elicit cross-neutralizing antibodies against EV71 virus subtypes. These findings demonstrate that the high-MOI production approach is significantly correlated with FP productivity, thereby improving the cross-neutralization efficacy of an EV71 vaccine and providing the basis for a better vaccine design against widespread EV71 viruses.

## Introduction

Enterovirus 71 (EV71), which belongs to the *Picornaviridae* family, is an icosahedral non-enveloped single-stranded RNA virus. During its replicative life cycle, EV71 can simultaneously generate fully infectious RNA-containing virions and immature empty particles. Virion assembly is initiated through cleavage of the viral RNA-encoded P1 polyprotein to generate the VP0 (36 kDa), VP1 (32 kDa) and VP3 (27 kDa) capsids, which further self-assemble into empty protein shells, also known as procapsids [1, 2]. Due to a lack of genomic RNA and the presence of uncleaved VP0, the empty protein shell is thought to be a putative assembly intermediate that has yet to undergo maturation. Following interactions between the viral RNA genome and capsid proteins, VP0 of the empty protein shell is autocatalyzed to be processed into VP2 (28 kDa) and VP4 (8 kDa), leading to mature virions [3, 4]. The cryo-electron microscopy structure of EV71 showed that the mature virions encapsidating the infectious RNA genome, called full particles (FPs), exhibit a compact shell morphology, whereas the population of immature empty protein shells, called empty particles (EPs), display an expanded particle morphology [5].

EV71 was recognized in 1997 as a major agent causing hand-foot-and-mouth disease (HFMD), which results in severe neurological illnesses and death in infants and young children in the Asia-Pacific region [6, 7]. Currently, vaccination is the most cost-effective way to prevent EV71 infection and related diseases. However, the worldwide co-circulation of several EV71 subgenotypes [8–17] might compromise the efficacy of currently licensed vaccines or vaccines in development based on monovalent whole-virus vaccines. Hence, the effective prevention and control of non-vaccine strain-associated diseases have become a challenge, and there is an urgent need to develop a broadly protective EV71 vaccine. Recently, clinical studies have implied that different EV71 genotype sublineages may share similar epitopes, eliciting the cross-strain neutralization against other non-vaccine strains [18, 19]. To improve vaccine design by enhancing the breadth and magnitude of neutralizing antibody protection, the immunological roles of critical vaccine components should be intensively investigated.

Consistent with EV71 replication in natural systems, the production of EV71 virus vaccine in bioreactors with a suspended microcarrier system yields both FP and EP particles [20]. A previous mouse vaccination study showed that FPs derived from the B4(E59) strain are more potent antigens than EPs and confer superior immunogenic efficacy in the neutralization of homologous virus [21]. However, it is unclear how FPs and EPs, the two key EV71 vaccine components, mediate the immune response to elicit cross-strain neutralizing antibodies against viruses.

Herein, we examined whether the virus input (multiplicity of infection; MOI), a key parameter of FP and EP generation dynamics, substantially affects vaccine productivity and efficacy in a suspended microcarrier production system. To better understand the immune responses induced by individual FPs and EPs, antisera prepared using different vaccine combinations were employed to characterize the neutralization breadth against EV71 viruses and to evaluate immune suppression with antigen competition. We also verified the specificities of anti-FP and anti-EP by competitive ELISA and defined FP as the critical vaccine component mediating the majority of viral neutralization efficacy, based on an antibody-depleted microneutralization assay. Finally, we performed a subunit protein adsorption assay to block the effective neutralizing antibodies induced from the two distinct EV71 particles and successfully identified the FP VP1 subunit as the major immunogen conferring wide-range neutralizing efficacy against EV71 strains. This study not only demonstrates the critical role of the mature EV71 virion in eliciting VP1-specific wide-spectrum cross-neutralizing immunity against EV71 viruses but also provides directions for improving the immunogenicity and efficacy of the current EV71 vaccine.

## Materials and methods

### Ethics statement

All animal experiments were conducted in accordance with the guidelines of the Laboratory Animal Center of the National Health Research Institutes (NHRI), Taiwan. The animal-use protocols were reviewed and approved by the NHRI Institutional Animal Care and Use Committee (Approved protocol no. NHRI-IACUC-101006-A). All of the tested animals were euthanized by 100% CO_2_ inhalation for 5 minutes followed by cervical dislocation to minimize the animals suffering after the completion of the experimental protocol (according to the guidelines of approved animal-use protocols; protocol no. NHRI-IACUC-101006-A).

### Cell and virus strains

African green monkey kidney cell lines (Vero cell) purchased from American Type Culture Collection (ATCC) and CGMP-certificated working virus bank (WVB) of the EV71 B4(E59) were obtained by technology transfer from National Health Research Institutes (NHRI), Taiwan, as described previously [20]. The EV71 virus subtypes, including 0242-TW86 (B1), SK-EV006 (B3), N2838-TW03 (B5), N1757-TW05 (C4A), and N3340-TW02 (C4B), were obtained from the National Health Research Institutes (NHRI), and EV71 C4(E36) was obtained from the Centers for Disease Control, Taiwan.

### EV71 virus production at different multiplicities of infection (MOIs)

The expansion of Vero cells from the T-flask to spinner flasks was described previously [20]. A 3-L spinner flask containing 3 g/L microcarrier (Cytodex-1, GE) was used for Vero cell growth, with stirring at 25–40 rpm in a 37°C, 5% CO_2_ incubator. When the cell density reached 8×10^5^ cells/mL, the mixture of cells and microcarriers was transferred and separated equally into four individual 1-L spinner flasks. Subsequently, the medium was replaced with fresh medium, and the cells were inoculated with ten-fold serial dilutions of EV71 B4(E59) virus stock to give MOIs of 10^−1^, 10^−2^, 10^−4^, or 10^−6^ pfu per cell. Daily sampling was performed to determine the virus titers at various time points post-infection. The endpoint of EV71 culture was the plateau of virus growth, as indicated by cytopathic effects (CPEs) greater than 90%. The virus titers were determined using a tissue culture infectious dose (TCID_50_) assay in Vero cells according to the Reed—Muench method [22].

### Purification of EV71 particles from varying MOI cultures

The purification of EV71 particles from the different MOI cultures was conducted as follows. The harvested culture supernatant was concentrated by 100 kDa tangential flow filtration (TFF) (EMD Millipore, USA) and partially purified by ultracentrifugation through a 20% sucrose cushion at 25,000 rpm in a SW28 rotor for 3 h at 4°C. The resulting virus pellets were resuspended in PBS buffer, further applied to a 20-30-40-50% sucrose density gradient and centrifuged at 35,000 rpm, 4°C for 2.5 h using a SW41 ultracentrifuge. The fractions were collected from the bottom of the gradients, and their sucrose concentrations were measured. The individual fractions from the varying MOI tests were resolved by SDS-PAGE analysis. Fractions 6 to 13 with sucrose concentrations ranging from 45% to 30% exhibited full particle (FP)-enriched and empty particle (EP)-enriched EV71 antigens. After pooling, dialysis against PBS, and inactivation with formalin, the purified EV71 particles produced from the different MOI tests were further used for yield evaluation (ELISA), mouse immunization to assess efficacy, and transmission electron microscopy (TEM) observations to quantify the FP/EP ratio. For virus inactivation, the samples prepared above were treated with 1:4000 (v/v) formalin and incubated at 37°C for 6 days as described previously [23].

### Electron microscopy of EV71 FPs and EPs

Three micrograms of purified EV71 particles derived from cell cultures infected with different virus MOIs was placed on a 200-mesh copper grid (Electron Microscopy Sciences) and stained with 2% methylamine tungstate (TED PELLA INC.). Micrographs were obtained by electron microscopy (FEG-TEM, FEI Tecnai G2 TF20 S-TWIN). Typically, the micrographs captured between 80–180 particles per image, providing a sufficient number of particles to determine morphology and numerical statistics for each sample. Approximately 10 TEM fields from each sample were randomly selected to count the total number of FPs and EPs. The statistics for the percentages of FPs and EPs among the total EV71 particle population are presented in a grouped stacked column plot.

### Animal immunization and specific FP/EP antisera preparation

Each dose of EV71 vaccine purified from different MOI virus cultures was mixed with 300 μg/mL Al(OH)_3_ in PBS buffer. Groups of BALB/c mice were intramuscularly immunized twice at days 0 and 14 and then bled at day 28 post-immunization to collect serum samples for microneutralization. To evaluate the neutralizing antibodies elicited by the FPs and EPs against the EV71 subtypes, the two distinct types of EV71 particles were highly purified and separated by continuous ultracentrifugation as described previously [20]. For evaluation of antigen competition, mouse antisera raised from EV71 vaccines composed of different FP and EP ratios were applied. To obtain sufficient FP and EP antisera for the cross-neutralization experiments, competitive ELISAs, and antigen/protein adsorption assays, New Zealand White (NZW) rabbits (3 months old, n = 3) were immunized by intramuscular injection into the quadriceps with 2 μg of Al(OH)_3_-adjuvanted FP or EP at days 0 and 21 and then bled at day 35 for serum collection.

### Competitive ELISA

FP or EP antisera containing specific antibodies were diluted 50-fold and then added to bovine serum albumin (BSA), B4(E59), C4(E36), FP, and EP competitors at different concentrations. This mixture was allowed to incubate overnight at 4°C. As a control and baseline for detection, phosphate-buffered saline containing 0.05% Tween 20 (PBST) was used in place of the antisera-competitor mixture. A 96-well microplate was precoated by incubation with EV71 B4, FP, and EP antigens (0.1 μg/well) diluted in PBS overnight at 4°C, followed by washing three times with PBST and blocking with 1% BSA in PBS buffer. The antisera-competitor samples were then added to the precoated plate, and after incubation at 37°C for 1 h, the wells were washed as described above. Antibodies that bound to the coated antigens were traced by goat anti-rabbit secondary antibodies conjugated with horseradish peroxidase (Cat.111-035-144; Jackson Immuno Research, USA). After incubation with the TMB substrate (Sigma), sulfuric acid was added to stop the reaction. Finally, the absorbance was measured at 450 nm (A_450_nm) with a Multi-Detection Microplate Reader (Synergy HT, Bio-Tek).

### Recombinant EV71 capsid protein preparation

The VP0 (amino acids 1–323), VP1 (amino acids 566–862), VP2 (amino acids 70–323), and VP3 (amino acids 324–565) subunits of the EV71 polyprotein derived from the human EV71 B4(E59) virus (GenBank: JN874551.1) were expressed in *Escherichia coli* BL21 cells (DE3) using the pTBSG1-his vector according to a previously published protocol [24]. Soluble maltose-binding protein (MBP), MBP-VP0, MBP-VP1, MBP-VP2, and MBP-VP3 proteins were purified by a Ni-NTA column and the protein concentrations were quantified via microBCA assays (Micro BCA Protein Assay Kit) for western blotting and neutralizing antibody depletion experiments.

### Depletion of viral-specific neutralizing antibodies

Each highly purified FP or EP of EV71 antigen (10 μg/well) and the MBP, MBP-VP1 or MBP-VP2 proteins (50 μg/well) were separately coated on tissue culture plates (12-well) overnight at 4°C. After blocking with 3% BSA and washing with PBS, the rabbit antisera elicited by EV71 B4(E59) FPs or EPs were individually incubated with the precoated antigens or MBP-fusion proteins for 1hr at 25°C to deplete the viral-specific neutralizing antibodies against FP, EP, VP1, or VP2. The same antisera were depleted in parallel with MBP or BSA as a control. Repeated depletion cycles were performed four times. The commercial Enterovirus 71 VP1 neutralizing antibody against EV71 genotype C4 (VP1/C4; 40013-H136, Sino biological Inc.) and VP2-specific MAB979/Anti-Enterovirus 71 antibody (MAB979; Merck Millipore) were treated with the same process to deplete the anti-VP1 and anti-VP2 antibodies. Anti-VP1 or anti-VP2 depletions were validated by the titer reductions in anti-FP pre-adsorbed with MBP-VP1 or MAB979 pre-adsorbed with MBP-VP2 compared to their MBP pre-adsorbed control groups (S1D–S1I Fig). The antisera with/without depletion of FP, EP, VP1, and VP2 antibodies were further applied for microneutralization.

### Microneutralization

Neutralizing antibodies against EV71 virus subtypes were evaluated by a microneutralization assay as described previously [20]. Viral plaques were stained with the MAB979 antibody, and the resulting plaques were counted by eye under a microscope. Microneutralization titers were calculated from the average of triplicate sample wells by extrapolating the inverse dilution of serum that produced a 100% reduction in virus by comparing the total number of plaques observed in PBS serum and vaccination serum samples. Microneutralization of the MAB979 mAb in this study was performed using a TCID_50_ assay in Vero cells according to the Reed—Muench method to avoid cross-reactivity with plaque staining [22]. The virus neutralization titers conferred by the tested antisera pre-adsorbed with BSA or MBP against individual viruses were set as 100% normalized neutralization. The neutralization inhibition value was calculated as {1- preabsorbed antisera neutralization (%) / 100%}×100%.

### Statistical analysis

Significant differences between two groups were statistically computed by applying the *t*-test using GraphPad Prism software Version 6.0 (GraphPad Software, USA).

## Results

### Multiplicity of infection (MOI) affects EV71 virus production in a suspended microcarrier system

**Fig 1:**
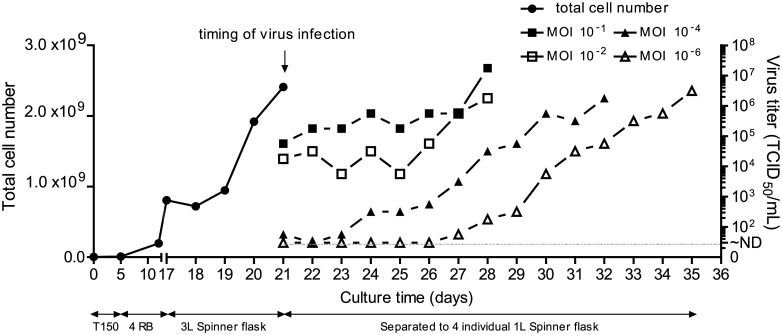
Different MOIs affect EV71 B4(E59) virus production in a suspended microcarrier culture system. Profile of Vero cell growth and EV71 amplification at different MOIs.

To investigate whether the MOI affects EV71 virus production, Vero cells were prepared in a 3-L spinner flask to maintain a consistent environment for further EV71 infection with different MOIs. A typical profile of cell growth is shown in Fig 1. When the culture in the 3-L spinner flask grew to a cell density of approximately 8 × 10^5^ cells/mL after 4 days, the culture was equally divided into four 1-L spinner flasks (S2a, S2c, S2e and S2g Fig), followed by EV71 virus inoculation at different MOIs of 10^−1^, 10^−2^, 10^−4^, and 10^−6^ (Fig 1). The virus production in the individual spinner flasks with MOIs of 10^−1^ and 10^−2^ reached maximal titers of 10^7.25^ to 10^6.25^ TCID_50_/mL, and the cells exhibited 95% cytopathic effects (cells detached from microcarrires) at day 7 post-infection (S2b and S2d Fig). At the lower MOIs of 10^−4^ and 10^−6^, the virus cultures achieved maximal titers ranging from 10^6.25^ to 10^6.5^ TCID_50_/mL at the harvest endpoint on days 10 and 14 post-infection, respectively (S2f and S2h Fig). Applying a high-MOI strategy for EV71 vaccine production may therefore be one of the most effective ways to shorten the upstream process and obtain high-titer virus cultures.

### Purification and characterization of EV71 particles from virus cultures with different MOIs

**Fig 2:**
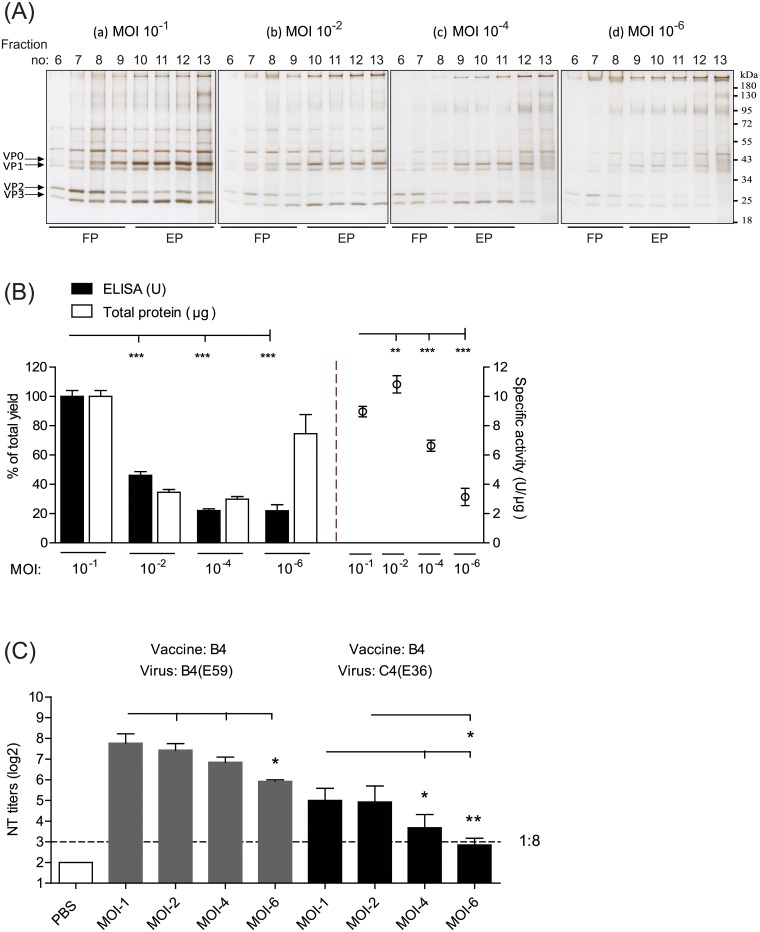
Productivity, specific activity, and immune potency of EV71 vaccines produced from different MOI cultures. (A) Purification of EV71 antigens from different MOI cultures by sucrose density gradient ultracentrifugation (SDG) and identification of the viral particle-enriched fractions using SDS-PAGE analysis with silver staining. (a)-(d). EV71 cultures at different MOIs as indicated above. The lines labeled with FP and EP represent the distributed fractions of the infectious full particles and defective empty particles, respectively. The molecular weights of VP0, VP1, VP2, and VP3 are indicated. The molecular weight marker is shown on the right. (B) The ELISA yield, total protein, and specific activity of EV71 vaccines purified from different MOI cultures. After SDG purification, the EV71 particle-enriched fractions (fractions 6–13) were subsequently pooled and inactivated for productivity and quality evaluation. (C) Viral neutralization titers against the B4(E59) and C4(E36) subgenotypes elicited by EV71 vaccines from different MOI preparations. Significant differences between vaccine groups are indicated with the following symbols: *, *p*<0.05; **, *p*<0.01; and ***, *p*<0.001.

The EV71 viruses produced from varying MOIs were harvested, separated from the cell debris and microcarriers, and concentrated by a tangential flow filtration (TFF) system. Subsequently, the concentrated viruses were purified by sucrose density gradient ultracentrifugation, and the protein profiles of the fractions were distinguished through SDS-PAGE analysis (Fig 2A). Silver staining revealed that the EV71 viral subunits (e.g., VP0, VP1, VP2, and VP3) co-localized and appeared to be restricted to fractions 6 to 13 (Fig 2A). For the virus cultures with MOIs of 10^−1^ and 10^−2^, fractions 6–9 and fractions 10–13 obtained from sucrose density gradient sedimentation appeared to be consistent with FP-enriched and EP-enriched EV71 antigens, respectively (Fig 2A, a-b). Similarly, the FP-enriched and EP-enriched antigens from the virus cultures with of MOIs 10^−4^ and 10^−6^ exhibited a relatively narrow distribution, in fractions 6–8 and 9–11, respectively (Fig 2A, c-d).

To compare the vaccine productivity and efficacy yields from varying MOIs, fractions 6 to 13 were pooled and treated with a final concentration of 0.025% formalin. The EV71-specific antigen and total protein yield in the vaccine bulk from the four groups were evaluated using Q-ELISA [25] and the microBCA method. The Q-ELISA is defined as a quantitative enzyme linked immunosorbent assay to determine the concentration of the EV71 VP2 antigen throughout the production cycle, particularly during the upstream harvest, downstream purification and viral inactivation steps [25]. The overall recovery yield of the Q-ELISA unit and the protein content obtained from the 10^−1^ MOI formulation were set as 100%±4.0. Compared to the MOI 10^−1^ group, the MOI 10^−2^, MOI 10^−4^, and MOI 10^−6^ formulations exhibited approximately 46%±2.5, 22%±1.3, and 22±4.1% retention of the Q-ELISA unit, respectively (Fig 2B, *left*, black column). The total protein yield from the MOI 10^−2^, MOI 10^−4^, and MOI 10^−6^ groups was obviously decreased, with 35%±1.9, 30%±1.7, and 75%±13.1 retention of the protein content, respectively, compared with the MOI 10^−1^ group (Fig 2B, *left*, white column). In accordance with the results shown in Fig 2B (*left*), the specific activities (U/μg) determined by dividing VP2-specific ELISA units by micrograms of protein were 9.0±0.36, 10.8±0.59, 6.6±0.38, and 3.1±0.59 for the MOI 10^−1^, MOI 10^−2^, MOI 10^−4^ and MOI 10^−6^ groups, respectively (Fig 2B, *right*).

To test whether the magnitude of the neutralizing response correlated with the Q-ELISA unit, groups of BALB/c mice (6–8 weeks old, n = 5) were immunized twice with 1 μg of inactivated EV71 vaccine from different MOI preparations on days 0 and 14. At 28 days post-immunization, mouse antisera were collected to determine viral neutralization titers (NT titers) against the cognate B4(E59) and C4(E36) subtype viruses. Mice immunized with EV71 vaccines from the MOI 10^−6^ to MOI 10^−1^ groups developed protective NT titers ranging from 1:61 to 1:218 against the homotype B4(E59) strain, suggesting that the NT titer magnitude might positively be correlated with the specific activity of EV71 vaccine (Fig 2C, *left*). For C4(E36) cross-strain neutralization, only mice immunized with EV71 vaccines derived from the MOI 10^−1^ and MOI 10^−2^ groups achieved substantial NT titers (1:29~1:24) (Fig 2C, *right*). In the MOI 10^−4^ and 10^−6^ groups, a lower cross-neutralizing response was induced against the C4(E36) virus (NT titers: 1:7~1:11) compared with the higher-MOI groups (higher MOIs vs. MOI 10^−4^, *p*<0.5; higher MOIs vs. MOI 10^−6^, *p*<0.01) (Fig 2C). These results suggested that the efficacy of the EV71 vaccine in skewing the immune response toward other EV71 subtypes might be determined by high-MOI-dependent production.

**Fig 3:**
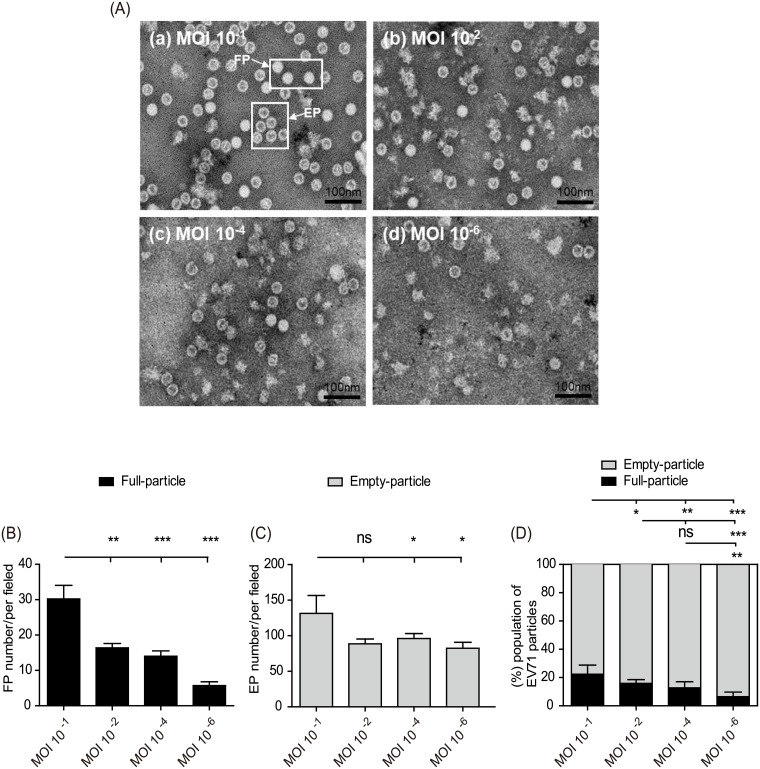
Characterization of FP and EP contents in EV71 vaccines with different production MOIs by transmission electron microscopy. (A) Pooled viral particles from the vaccines prepared from (a) MOI 10^−1^, (b) MOI 10^−2^, (c) MOI 10^−4^, and (d) MOI 10^−6^ cultures were observed by TEM. The morphologies of the FPs and EPs are indicated by arrows. The total numbers of viral particles in the different MOI samples were statistically counted, and the average sums of the FPs (B) and EPs (C) per field were plotted. (D) Percent normalization of FP and EP content to the total particles from each EV71 vaccine prepared from cultures with different MOIs. The total particle number for each EV71 vaccine produced using different MOIs was set as 100%. Significant differences between groups are indicated with the following symbols: *, *p*<0.05; **, *p*<0.01; and ***, *p*<0.001. ns: no significant difference was present between the groups.

To investigate whether high viral MOIs may lead to a shift in the FP and EP populations during vaccine production, we examined the EV71 particle composition among the four vaccine groups by TEM. We found that the physical morphologies of the FPs or EPs were approximately 30–35 nm in diameter, consistent with a previous study (Fig 3A) [20]. Statistically counting the number of FPs and EPs in the EV71 vaccines with different MOIs revealed that the FP population was the minor component in the EV71 vaccine but significantly increased when the vaccine was produced with a high-MOI virus inoculation (Fig 3B). Furthermore, EPs were the predominant population among the EV71 vaccines throughout the low- to high-MOI production samples (Fig 3C). A stacked plot of the normalized FP and EP subpopulation percentages in the EV71 vaccines clearly showed that high-MOI production significantly increased the FP subpopulation compared with low-MOI production (Fig 3D). The results of mouse immunological studies and TEM analysis thus showed that high-MOI viral inoculation produced more FPs for an EV71 vaccine in a microcarrier bioreactor system and that the FP content may decide the vaccine cross-neutralization potency toward EV71 subgenotypes (Figs 2C and 3D).

### Characterization of neutralizing responses elicited by FPs and EPs

**Fig 4:**
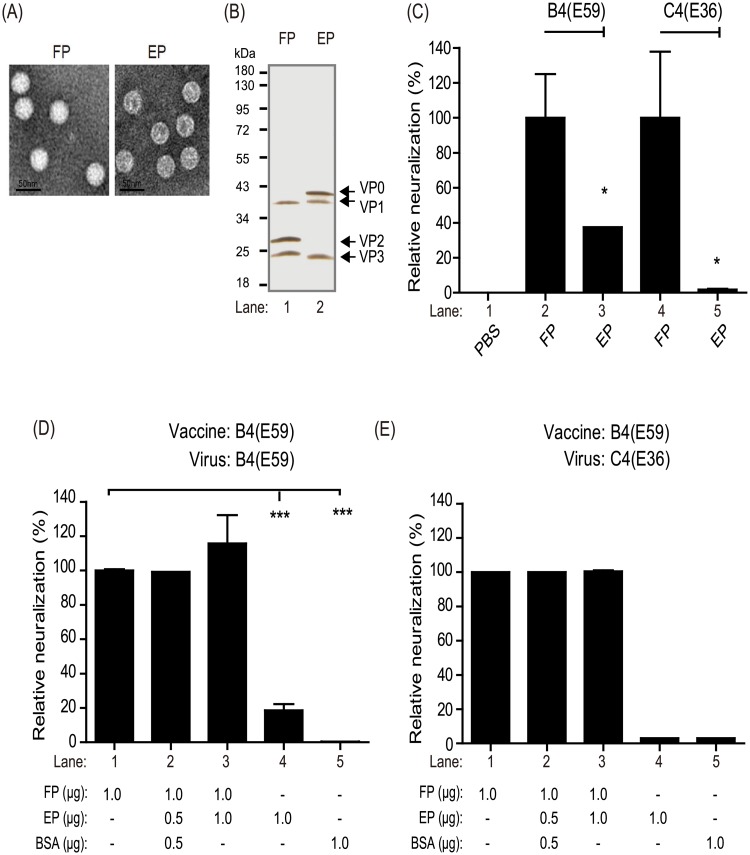
Virus neutralization efficacy and antigenic competition responses for FPs and EPs. Quality confirmation of purified FPs and EPs based on TEM observations (A) and SDS-PAGE analysis with silver staining (B). The molecular weights of VP0, VP1, VP2, and VP3 are indicated. (C) Measurement of neutralizing efficacy against the B4(E59) or C4(E36) subgenotype EV71 provided by anti-FP and anti-EP. The neutralizing titers raised from FPs were set as 100% normalized neutralization. The percent reduction in neutralization conferred by anti-EP was calculated. Evaluation of antigenic competition between EV71 vaccines composed of different FP and EP ratios. The neutralizing titer raised from 1 μg of FP was set as 100% normalized neutralization. The B4(E59) (D) or C4(E36) (E) neutralization conferred by antisera from manually prepared vaccines was calculated. Significant differences between groups are indicated by the following symbols: *, *p*<0.05; **, *p*<0.01; and ***, *p*<0.001.

To examine the immunological roles of the two EV71 particles, the purified FPs and EPs derived from B4(E59) were used to individually immunize rabbits for the production of specific anti-FP and anti-EP antibodies (Fig 4A and 4B). Anti-FP had higher geometric mean NT titer = 1:20,646 than anti-EP (geometric mean NT titer = 1:8,192, *p*<0.05) against the B4(E59) virus. To normalize the data, the antibody response elicited by the FPs against the B4(E59) virus was set as 100%±25.0 neutralized; by comparison, anti-EP showed only 39%±0 neutralization (Fig 4C, B4(E59)). Interestingly, the cross-neutralizing responses against the C4(E36) virus conferred by the FPs and EPs differed greatly. Compared to anti-FP (geometric mean NT titer = 1:256), which resulted in 100%±37.8 cross-neutralization, anti-EP was unable to provide cross-strain neutralizing efficacy (geometric mean NT titer = 1:5) and presented ≤2%±0.4 normalized neutralization against C4(E36) (Fig 4C, lane 4 vs. lane 5).

As noted previously, for EV71 vaccine production in the microcarrier system, most particles were EPs (80%), and real infectious FPs represented an average of only 10–20% of the total particles (Fig 3D). We next investigated whether the EPs exhibited antigen-competition effects suppressing the immune response elicited by FPs. EV71 vaccines containing FPs combined with different amounts of EPs or bovine serum albumin (BSA) were manually prepared for the mice immunization study. Five groups of mice were immunized with 1.0 μg of FP, 1.0 μg of FP + 0.5 μg of EP + 0.5 μg of BSA, 1.0 μg of FP + 1.0 of μg EP, 1.0 μg of EP alone or 1.0 μg of BSA alone, respectively. As shown in Fig 4D, the antisera elicited by 1 μg of FP had the highest NT titer against B4(E59) (geometric mean NT titer = 1:1,024), which was set as 100%±0 neutralization efficacy; the percent neutralization for each formulation was normalized to the efficacy provided by 1 μg of FP. The antisera from the 1.0 μg of FP + 0.5 μg of EP + 0.5 μg of BSA (geometric mean NT titer = 1:1,024) or 1.0 μg of FP + 1.0 μg of EP (geometric mean NT titer = 1:1,172) groups exhibited equivalent 100%±0 or 117%±17 relative neutralizing activities and did not differ significantly from the control group (Fig 4D, lanes 1–3). A similar phenomenon was observed in the C4(E36) cross-neutralization results (Fig 4E, lanes 1–3; 1 μg of FP had the highest NT titer against C4(E36) (geometric mean NT titer = 1:128)). The antisera from the mice immunized with EP or BSA provided only 19.0%±3.6 (geometric mean NT titer = 1:181) or 0.4%±0 (geometric mean NT titer = 1:4) neutralization efficacy against B4(E59), respectively, compared to the mice immunized with identical dosages of FPs (Fig 4D, lanes 1, 4, and 5). These results demonstrated that the immune responses induced by the FPs were not obviously affected by the addition of additional EPs or BSA to elicit neutralizing antibodies, suggesting that EPs or BSA may not competitively suppress the FP-induced immune response in mice. Taken together, these findings indicate that FP in the EV71 vaccine plays a significant role in eliciting a neutralizing antibody response against EV71 subtypes, whereas EPs provide only low humoral immunity against the B4(E59) virus and are not involved in cross-neutralizing strains with other genotypes or immune interference (Fig 4C–4E).

### Characterization of anti-FP and anti-EP binding specificity

**Fig 5:**
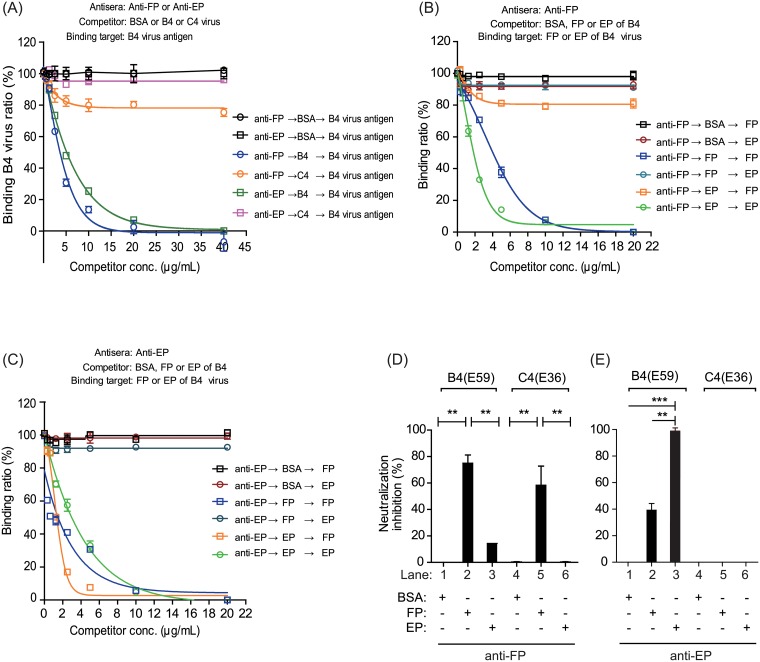
The binding specificities of anti-FP and anti-EP correlate with virus neutralization efficacy. (A) The binding specificities of anti-FP and anti-EP to the target virus B4(E59) were examined by adding virus antigens from B4(E59) or C4(E36) as competitors in a competition ELISA. The binding efficiencies of anti-FP (B) and anti-EP (C) to FP or EP from the B4(E59) virus were evaluated by competitive ELISA assay. The antisera, competitors, and binding targets are indicated. Inhibition of viral neutralization by anti-FP or anti-EP against the B4(E59) (D) or C4(E36) (E) virus was examined by FP- or EP-adsorption experiments. BSA was used as the non-EV71 competitor/antigen, as a negative control in the competitive ELISAs and viral neutralization inhibition assays. The substantial neutralizing titers (≥1:128) conferred by BSA-pre-adsorbed anti-FP or anti-EP against the viruses were set as 100% normalized neutralization. The increase in the neutralization inhibition percentage was measured by pretreating anti-FP and anti-EP with FP or EP adsorption. Significant differences between groups are indicated by the following symbols: *, *p*<0.05; **, *p*<0.01; and ***, *p*<0.001. ns: no significant difference was present between the groups.

To further characterize the antisera raised against FPs and EPs, we investigated the specificity of anti-FP and anti-EP using a competitive ELISA in which competitors inhibit antisera to bind the target antigens. Competition studies revealed that the purified B4(E59) virus not only blocked anti-FP but also inhibited anti-EP from binding to homologous B4(E59) virus (Fig 5A, blue and green). Purified C4(E36) virus reduced the binding capacity of anti-FP by 20% but did not block the anti-EP to react with homologous B4(E59) virus compared with the antisera blocked by BSA, suggesting that only anti-FP can cross-react with heterologous virus (Fig 5A, orange vs. purple).

To better understand the binding specificity of anti-FP and anti-EP, purified FPs or EPs from EV71 were as used as competitors to block target antigen binding by anti-FP and anti-EP (S3 and S4 Figs). The results showed that the purified FPs from EV71 efficiently blocked anti-FP from binding to FP (Fig 5B, blue square; S3 Fig). Furthermore, pure EPs reduced the binding capacity of anti-FP to react with FPs by 20% compared with the antisera blocked by BSA (Fig 5B, orange square vs. black square; S3 Fig). Although EPs purified from EV71 completely inhibited the binding of EPs by anti-FP, this specific interaction was not obviously blocked by FPs (Fig 5B, green and deep-blue circles). We further examined the anti-EP binding epitopes on the EV71 particles and found that purified FPs from EV71 only blocked anti-EP from binding to FPs and did not obviously reduce the binding capacity to react with EPs (Fig 5C, blue square and deep-blue circle vs. red circle; S4 Fig). By contrast, the purified EPs from EV71 efficiently inhibited anti-EP from binding to either EPs or FPs (Fig 5C, green circle and orange square; S4 Fig). These results indicated that anti-FP likely binds to FPs (80%), whereas only a small population of anti-FP binds to EPs (20%). The reverse results showed that the majority of anti-EP preferentially binds to EPs (90%), whereas less binds to FPs (10%), suggesting that anti-EP is sensitively blocked by EPs and does not efficiently bind FPs (Fig 5C, orange square), resulting in poor EV71 B4(E59) virus neutralization, as observed in Fig 4C.

To determine whether the *in vitro* binding capacity also mediates the virus neutralization efficacy, the antisera were preabsorbed with FPs or EPs from EV71 to evaluate virus-neutralizing antibody titers. Anti-FP preabsorbed by FPs inhibited approximately 75%±3.6 and 60%±8.3 of the neutralization effect for homologous and heterologous EV71 subtypes, respectively (Fig 5D, lanes 2 and 5); however, anti-FP preabsorbed by EPs only blocked 15%±0 and 0%±0 of the efficacy to neutralize the homologous and heterologous EV71 viruses, respectively (Fig 5D, lanes 3 and 6). By contrast, anti-EP preabsorbed by FPs or EPs greatly reduced the neutralizing titers against the homologous EV71 virus by 37.5%±6.2 and 99%±0.4, respectively, compared with the BSA-preabsorbed control group (0%) (Fig 5E, lanes 1–3). Anti-EP did not bind and confer cross-neutralizing titers to C4(E36) and thus exhibited no inhibition in antisera preadsorbed by either FPs or EPs (Figs 4C and 5A and 5E, lanes 5 and 6). These findings suggested that the antigenic binding capabilities of anti-FP and anti-EP correlate with their viral neutralization efficacies (Fig 5). Taken together, these results indicated that antisera from FPs have great potential to confer wide-range neutralizing immunity against homologous and heterologous EV71 subtypes, whereas anti-EP only provides minor neutralizing efficacy against homologous virus (Figs 4C and 5).

### An FP-elicited VP1-specific antibody confers cross-neutralizing efficacy against EV71 subgenotypes

**Fig 6:**
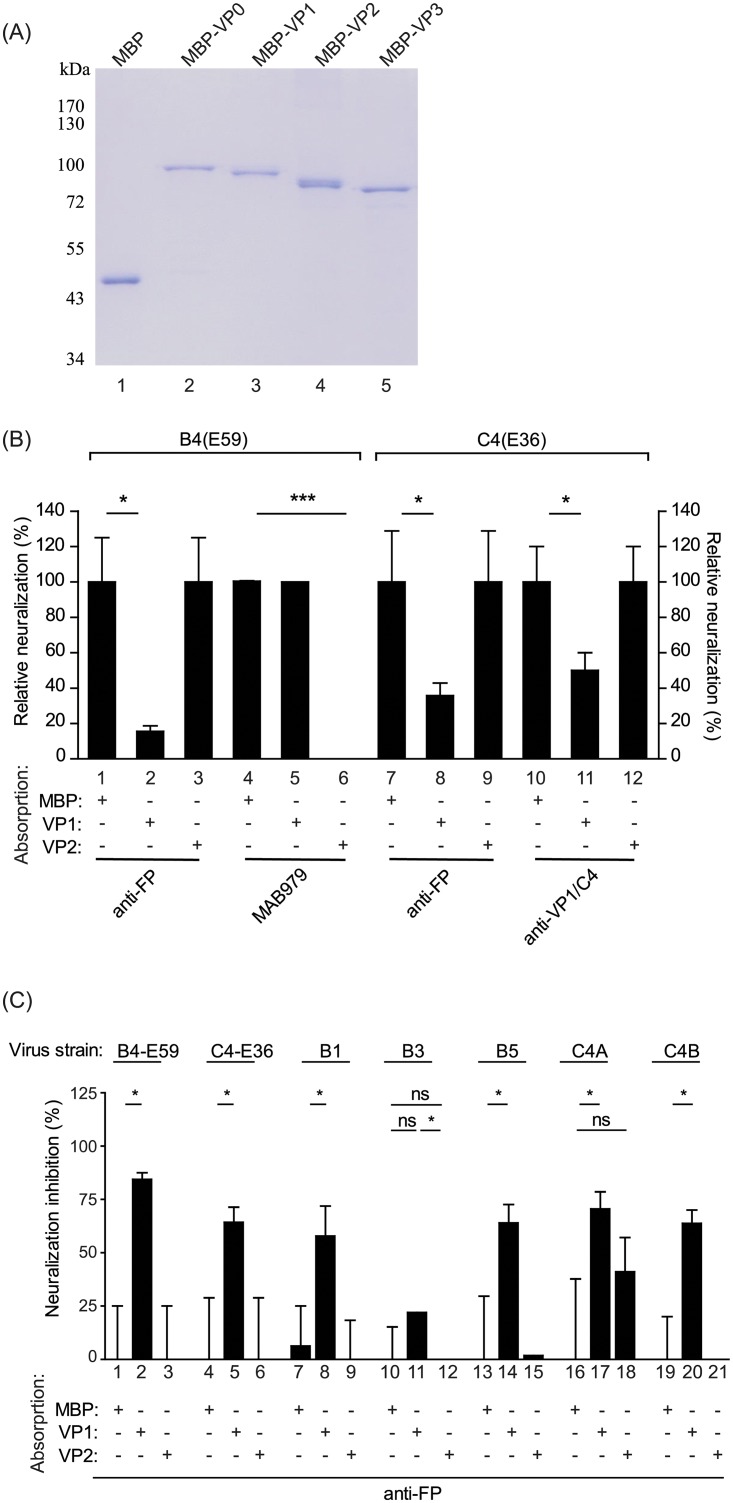
A VP1-specific antibody raised from FPs exhibits broad neutralization efficacy against EV71 subgenotype viruses. (A) The purity of maltose-binding protein (MBP) fusions with the VP0, VP1, VP2, and VP3 EV71 capsid proteins was confirmed by SDS-PAGE analysis with Coomassie blue staining. (B) The neutralization activities of protein-preabsorbed antisera or antibodies against the B4(E59) or C4(E36) virus were measured. The tested antisera are labeled. MAB979 is a monoclonal VP2-specific antibody that neutralizes the B4(E59) virus and was used as a positive control for MBP-VP2 adsorption. The anti-VP1/C4 monoclonal antibody confers neutralization activity against C4(E36) and was used as a positive control for MBP-VP1 adsorption. (C) Measurement of EV71 subgenotype neutralization inhibition by preabsorbed anti-FP. The neutralizing titer provided by anti-FP pre-adsorbed with MBP against individual viruses was set as 100% normalized neutralization. The increase in the percentage of neutralization inhibition conferred by anti-FP pre-adsorbed with MBP-VP1 or MBP-VP2 was calculated and normalized to the MBP-adsorption groups. The EV71 virus strains used for this experiment are labeled. Significant differences between groups are indicated by the following symbols: *, *p*<0.05; **, *p*<0.01; and ***, *p*<0.001. ns: no significant difference was present between the groups.

Based on our knowledge, it is unclear that which subunit proteins on FPs play a major role for eliciting the neutralized antibodies against EV71 viruses. To address that, the EV71 viral subunits VP0, VP1, VP2, and VP3 were prepared by individually fusing them with maltose-binding protein (MBP) to create MBP-VP0, MBP-VP1, MBP-VP2, and MBP-VP3, respectively, to improve protein solubility and stability. The purity of the protein samples was analyzed by SDS-PAGE with Coomassie blue staining (Fig 6A). The MBP alone, MBP-VP0, MBP-VP1, MBP-VP2, or MBP-VP3 proteins were used to absorb antisera raised from FPs. The preabsorbed antisera were then analyzed by western blotting to observe the specificity of the depleted antisera titers. The antibody titers against the VP1 subunit from FP antisera were reduced by adsorption with MBP-VP1 but not MBP or MBP-VP2 (S1D–S1F Fig). Furthermore, the VP2-specific MAB979 antibody recognized MBP-VP0, MBP-VP2, and VP0/VP2 from EV71 particles, and the corresponding signals decreased by approximately 50% when MBP-VP2-adsorbed MAB979 was used for detection (S1G–S1I Fig).

Anti-FP absorbed with MBP-VP1 exhibited a decrease in the relative neutralization percentage against B4(E59) of 85%±3.1 compared with the antisera absorbed with MBP-VP2 or MBP (Fig 6B, *Left*; anti-FP). By contrast, the neutralization of the B4(E59) virus by the VP2-specific MAB979 mAb decreased by 100%±0 after depletion by MBP-VP2 (Fig 6B, *Left*; MAB979). The other heterologous virus neutralization results showed that the anti-VP1/C4 mAb and antisera from FPs that were adsorbed by MBP-VP1 resulted in a significant suppression of C4(E36) virus neutralization by 50%±10.0 and 65%±7.1 (Fig 6B, *Right*; anti-VP1/C4 & anti-FP). These results suggested that different EV71 subgenotypes may share conserved epitopes on the FP VP1 subunit. Interestingly, even though anti-EP presented neutralizing antibody titers against the B4(E59) virus (geometric mean NT titer = 1:8,192), this antibody-mediated neutralization was not affected by MBP-VP1 or MBP-VP2 adsorption.

We next investigated whether the cross-neutralizing antibodies against EV71 viruses elicited by the VP1 subunit from FP. The anti-FP preabsorbed with MBP only, MBP-VP1 or MBP-VP2 were individually applied to the microneutralization assay against EV71 subgenotypes B1, B3, B5, C4A, and C4B. In contrast to the groups with anti-FP with MBP and MBP-VP2 adsorption, the group in which the VP1 antibody was depleted from anti-FP absorbed with MBP-VP1 showed apparent neutralization inhibition among EV71 viruses (Fig 6C). Although we observed that anti-FP preabsorbed with MBP-VP2 reduced the neutralization efficacy against C4A by 40%±16, the inhibition results did not differ significantly between the MBP-alone and MBP-VP2 adsorption groups (Fig 6C, C4A). These results provide evidence that the FP of EV71 plays an important role in eliciting a VP1-specific antibody that confers broad neutralizing efficacy against multiple EV71 virus subtypes.

## Discussion

The circulation and genomic recombination of EV71 subgenotype viruses has caused life-threatening epidemics and outbreaks in the Asia-Pacific region. Recent reports have shown that neutralizing antibodies elicited by the C4 vaccine strain can cross-react with the C2, C4, C5, B4 and B5 subgenotypes, whereas the B4(E59) vaccine subgenotype can also elicit neutralizing antibodies against the B1, B4, B5, and C4a subgenotypes [18, 19]. This cross-reactivity implies that different EV71 genotype sublineages may share conserved epitopes within the virus composition. Thus, understanding the mechanism underlying cross-strain neutralizing potential is essential to improve vaccine immunogenicity and efficacy.

In this report, we systematically investigated the effects of inocula with varying MOIs, which influence the dynamics of infectious FPs and defective EPs, on EV71 vaccine production in a serum-free microcarrier bioreactor system. The results showed that the high-MOI inocula effectively shortened the upstream manufacturing time to obtain high titers of EV71 virus (Fig 1). Furthermore, in contrast to the MOI 10^−4^ and 10^−6^ inocula, the EV71 vaccines produced from high MOIs of 10^−1^ and 10^−2^ exhibited higher productivity and better cross-neutralizing efficacy, which may improve the development of current EV71 vaccines (Fig 2). The statistical TEM results also showed that the higher-MOI inoculation for EV71 production resulted in a greater proportion of FPs in the EV71 vaccine composition (Fig 3). These results suggested that FPs may play a dominant role affecting vaccine efficacy and are responsible for improving efficacy against subgenotype strains, which has not been previously recognized.

To dissect the contributions of FPs and EPs to vaccine efficacy, the specific anti-FP or anti-EP antibodies produced from rabbits immunized with purified FPs or EPs were used for virus neutralization (Fig 4A and 4B). We found that antisera raised against either FPs or EPs exhibited neutralizing titers toward B4(E59). The FP-induced antisera showed 100% neutralization efficacy compared with the antisera raised against EP, which exhibited only 39% neutralization efficacy (Fig 4C, B4(E59)). The cross-neutralization results showed that anti-FP provided neutralizing titers against C4(E36), whereas anti-EP had no efficacy. These findings suggested that FPs play a dominant role in eliciting cross-neutralizing antibody immune responses against EV71 viruses (Fig 4C, C4(E36)).

Understanding the phenomenon of antigenic competition in multicomponent vaccines is important for optimizing the formulation for vaccine efficacy. We therefore further addressed whether any immunological interference occurred between FPs and EPs that would suppress the immune response for eliciting neutralizing antibodies. Our results showed that FPs play a dominant role in the magnitude of neutralizing antibody efficacy against EV71 virus subtypes and illustrated that EPs may not be effective for providing antibody-dependent cross-neutralization activity because they did not suppress the FP-eliciting neutralizing immune responses (Fig 4D and 4E).

We further characterized the specificity of anti-FP and anti-EP by competitive ELISA and observed a positive correlation between the antisera binding capacity and viral neutralization efficacy. Our results showed that both antisera were completely inhibited by the B4(E59) virus (Fig 5A). Interestingly, only anti-FP showed a partially reduced binding ability to B4(E59) when using the C4(E36) virus as a competitor. However, the anti-EP binding of B4(E59) was not affected by the C4(E36) virus (Fig 5A). These observations are consistent with the neutralization results, which showed that both antisera were capable of eliciting neutralizing antibodies against the B4(E59) homologous virus, whereas only anti-FP enabled cross-neutralization against the C4(E36) virus (Figs 4C vs. 5A). In addition to understanding the specific antigen-binding properties of anti-FP and anti-EP, we also determined that both antisera could recognize the epitopes on both FPs and EPs (Fig 5B and 5C). The largest difference between the two antisera was that anti-FP preferentially bound FP over EP (80% vs. 20%, Fig 5B, black square vs. orange square). Similarly, anti-EP preferentially bound EP, which was rarely suppressed by FPs (90% vs. 10%, Fig 5C, red circle vs. blue circle). More interestingly, the specific binding of anti-EP to FP was efficiently inhibited by EP, implying that anti-EP neutralization of the B4(E59) virus might be trapped by pre-binding with an excess of pre-existing EPs during virus replicative cycles, thus resulting in ineffective neutralization of EV71 virus (Figs 4C and 5C).

Furthermore, adsorption experiments demonstrated that most of the neutralizing antibodies were elicited by FPs because the neutralizing titers of anti-FP were efficiently depleted by FPs against the B4(E59) and C4(E36) viruses, in contrast to the effects of pre-adsorption with EPs (Fig 5D). Since anti-EP did not preferentially bind FPs and was sensitively blocked by EPs, its neutralizing efficacy against the B4(E59) virus was low (Figs 5C, 5E and 4C). Due to the lack of binding and neutralization of the C4(E36) virus with anti-EP (geometric mean NT titer <1:8), we were unable to observe any inhibition effects of the adsorption of anti-EP by FPs or EPs on C4(E36) neutralization (Figs 5A, 5E, and 4C). Taken together, these virus neutralization results correlated with the binding specificity of anti-FP and anti-EP observed in the competitive ELISA assays. These findings indicate that FPs have great potential to improve current EV71 vaccine efficacy by eliciting cross-neutralizing humoral immunity against EV71 virus subgenotypes.

To address which subunits may present major epitopes on FPs that elicit cross-neutralizing antibodies, we prepared MBP-fused VP0, VP1, VP2, and VP3 subunit proteins and performed western blot analysis by probing with antisera raised against the inactivated virus vaccine, FP, and the VP2-specific monoclonal antibody MAB979 (Fig 6A and S1 Fig). Our results showed that the inactivated EV71 virus vaccine-elicited neutralizing antibodies (anti-EV71) recognized the epitopes on the VP1, VP2, and VP3 subunit proteins, consistent with previous studies [26, 27] (S1A Fig). The FP antisera exhibited strong signals against the VP1 subunit, but very minor activity against the VP2 subunit from prepared protein samples (S1B and S1D Fig). The subunit protein adsorption study demonstrated that the neutralizing efficacy of FP antisera against B4(E59) and C4(E36) decreased by 85% and 65%, respectively, after VP1 antibody depletion, in contrast to VP2 or MBP adsorption (Fig 6B). These results for EV71 are similar to those obtained for poliovirus; a study in which virus-specific antibodies were raised in rats against the VP1, VP2, and VP3 capsid proteins showed that VP1 appears to be the most important neutralizing antigen for poliovirus inhibition [28].

Our previous study showed that an alum-adjuvanted B4(E59) vaccine protects infant Tg-mice against C4B (N3340-TW02) virus lethal challenge through a neutralizing antibody-dependent mechanism, rather than a cell-mediated immune response [20]. To investigate whether the FPs from EV71 elicit a VP1-specific antibody response against a wide range of EV71 viruses, anti-FP with protein adsorption was applied to a neutralization assay against a panel of EV71 virus subtypes. Neither VP2 nor MBP adsorption, anti-FP with VP1 adsorption inhibited more than 50% of the neutralization efficacy against the B4(E59), C4(E36), B1, B5, C4A, and C4B viruses (Fig 6C). The data reported here suggest that the FP VP1 subunit is the major immunogen conferring cross-neutralization humoral immunity against EV71 subtypes and pave the way for the development of better protective EV71 vaccines aimed at controlling widespread EV71.

## Supporting information

S1 Text Identification of viral subunits recognized by EV71 vaccine-, FP-, and EP-induced antisera. In contrast to the lack of reactivity against the MBP protein among all tested antisera, the EV71 virus vaccine-elicited antisera (anti-EV71) strongly recognized MBP-VP1 and the FP and EP VP1 subunits and showed minor reactivity against the MBP-VP0, MBP-VP2, MBP-VP3, FP VP2, and VP3 subunits (S1A Fig). The FP antisera exhibited strong reactive signals with MBP-VP1 and the FP and EP VP1 subunits (S1B Fig), demonstrating that anti-FP elicits a major VP1-specific antibody. However, the antisera from EV71 EPs showed extremely low reactivity against all MBP-tagged viral proteins and viral subunits in western blot analysis (S1C Fig). Furthermore, the binding titers of anti-EP to the EV71(B4) virus were equivalent to those of anti-FP, but anti-EP exhibited no reactivity against any of the MBP-fusion proteins in the ELISA assay. These results suggest that the antigenic epitopes in anti-EP are very different from those in anti-FP from the EV71 virus.
(DOC)

S1 Fig Western blot analysis of the binding specificity of anti-EV71, anti-FP and anti-EP. Protein samples of MBP, MBP-VP0, MBP-VP1, MBP-VP2, MBP-VP3, FPs and EPs from EV71 were separated on a 10% SDS-PAGE gel and transferred to a PVDF membrane for western blot analysis against the (A) anti-EV71 vaccine, (B) anti-FP, and (C) anti-EP. The binding titer reduction after protein adsorption treatment was confirmed by western blot analysis with probes consisting of anti-FP adsorbed with MBP (D), anti-FP adsorbed with MBP-VP1 (E), anti-FP adsorbed with MBP-VP2 (F), anti-MAB979 adsorbed with MBP (G), anti-MAB979 adsorbed with MBP-VP1 (H), and anti-MAB979 adsorbed with MBP-VP2 (I).
(PDF)

S2 Fig Photomicrographs of Vero cells on microcarriers. The Vero cells/microcarriers mixture was sampled from each 1-L spinner flask immediately before infection with EV71 virus (a, c, e, g) and before harvest (b, d, f, h) at varying MOIs.
(PDF)

S3 Fig Proposed mode of specificity for anti-FP binding to FP and EP in the competitive ELISA study. Rabbits were immunized with FPs to generate antibodies that recognized FPs or EPs. In the competitive ELISA, the binding specificity for FPs of anti-FP blocked by BSA was defined as 100% (a). The binding specificity for FP of anti-FP blocked by FP was calculated as 0% (b). The binding specificity for FP of anti-FP blocked by EP was calculated as 80% (c). The binding specificity for EP of anti-FP blocked by EP was calculated as 0% (d).
(PDF)

S4 Fig Proposed mode of specificity for anti-EP binding to FP and EP in the competitive ELISA study. Rabbits were immunized with EPs to generate antibodies that recognized FPs or EPs. In the competitive ELISA, the binding specificity for EP of anti-EP blocked by BSA was defined as 100% (a). The binding specificity for FP of anti-EP blocked by FP was calculated as 0% (b). The binding specificity for EP of anti-EP blocked by FP was calculated as 90% (c). The binding specificity for EP or FP of anti-EP blocked by EP was calculated as 0% (d).
(PDF)
