# Supplementary material for: The mature EV71 virion induced a broadly cross-neutralizing VP1 antibody against subtypes of the EV71 virus
Source: PLoS One. 2019 Jan 16;14(1):e0210553. doi: 10.1371/journal.pone.0210553 (PMC6334917; doi:10.1371/journal.pone.0210553)
Supplement: S1 Text — In contrast to the lack of reactivity against the MBP protein among all tested antisera, the EV71 virus vaccine-elicited antisera (anti-EV71) strongly recognized MBP-VP1 and the FP and EP VP1 subunits and showed minor reactivity against the MBP-VP0, MBP-VP2, MBP-VP3, FP VP2, and VP3 subunits (S1A Fig). The FP antisera exhibited strong reactive signals with MBP-VP1 and the FP and EP VP1 subunits (S1B Fig), demonstrating that anti-FP elicits a major VP1-specific antibody. However, the antisera from EV71 EPs showed extremely low reactivity against all MBP-tagged viral proteins and viral subunits in western blot analysis (S1C Fig). Furthermore, the binding titers of anti-EP to the EV71(B4) virus were equivalent to those of anti-FP, but anti-EP exhibited no reactivity against any of the MBP-fusion proteins in the ELISA assay. These results suggest that the antigenic epitopes in anti-EP are very different from those in anti-FP from the EV71 virus. (DOC) [file pone.0210553.s001.doc]

**S1 Text. Identification of viral subunits recognized by EV71 vaccine-, FP-, and EP-induced antisera.** In contrast to the lack of reactivity against the MBP protein among all tested antisera, the EV71 virus vaccine-elicited antisera (anti-EV71) strongly recognized MBP-VP1 and the FP and EP VP1 subunits and showed minor reactivity against the MBP-VP0, MBP-VP2, MBP-VP3, FP VP2, and VP3 subunits (S1A Fig). The FP antisera exhibited strong reactive signals with MBP-VP1 and the FP and EP VP1 subunits (S1B Fig), demonstrating that anti-FP elicits a major VP1-specific antibody. However, the antisera from EV71 EPs showed extremely low reactivity against all MBP-tagged viral proteins and viral subunits in western blot analysis (S1C Fig). Furthermore, the binding titers of anti-EP to the EV71(B4) virus were equivalent to those of anti-FP, but anti-EP exhibited no reactivity against any of the MBP-fusion proteins in the ELISA assay. These results suggest that the antigenic epitopes in anti-EP are very different from those in anti-FP from the EV71 virus.
