# Supplementary material for: The mature EV71 virion induced a broadly cross-neutralizing VP1 antibody against subtypes of the EV71 virus
Source: PLoS One. 2019 Jan 16;14(1):e0210553. doi: 10.1371/journal.pone.0210553 (PMC6334917; doi:10.1371/journal.pone.0210553)
Supplement: S1 Fig — Protein samples of MBP, MBP-VP0, MBP-VP1, MBP-VP2, MBP-VP3, FPs and EPs from EV71 were separated on a 10% SDS-PAGE gel and transferred to a PVDF membrane for western blot analysis against the (A) anti-EV71 vaccine, (B) anti-FP, and (C) anti-EP. The binding titer reduction after protein adsorption treatment was confirmed by western blot analysis with probes consisting of anti-FP adsorbed with MBP (D), anti-FP adsorbed with MBP-VP1 (E), anti-FP adsorbed with MBP-VP2 (F), anti-MAB979 adsorbed with MBP (G), anti-MAB979 adsorbed with MBP-VP1 (H), and anti-MAB979 adsorbed with MBP-VP2 (I). (PDF) [file pone.0210553.s002.pdf]

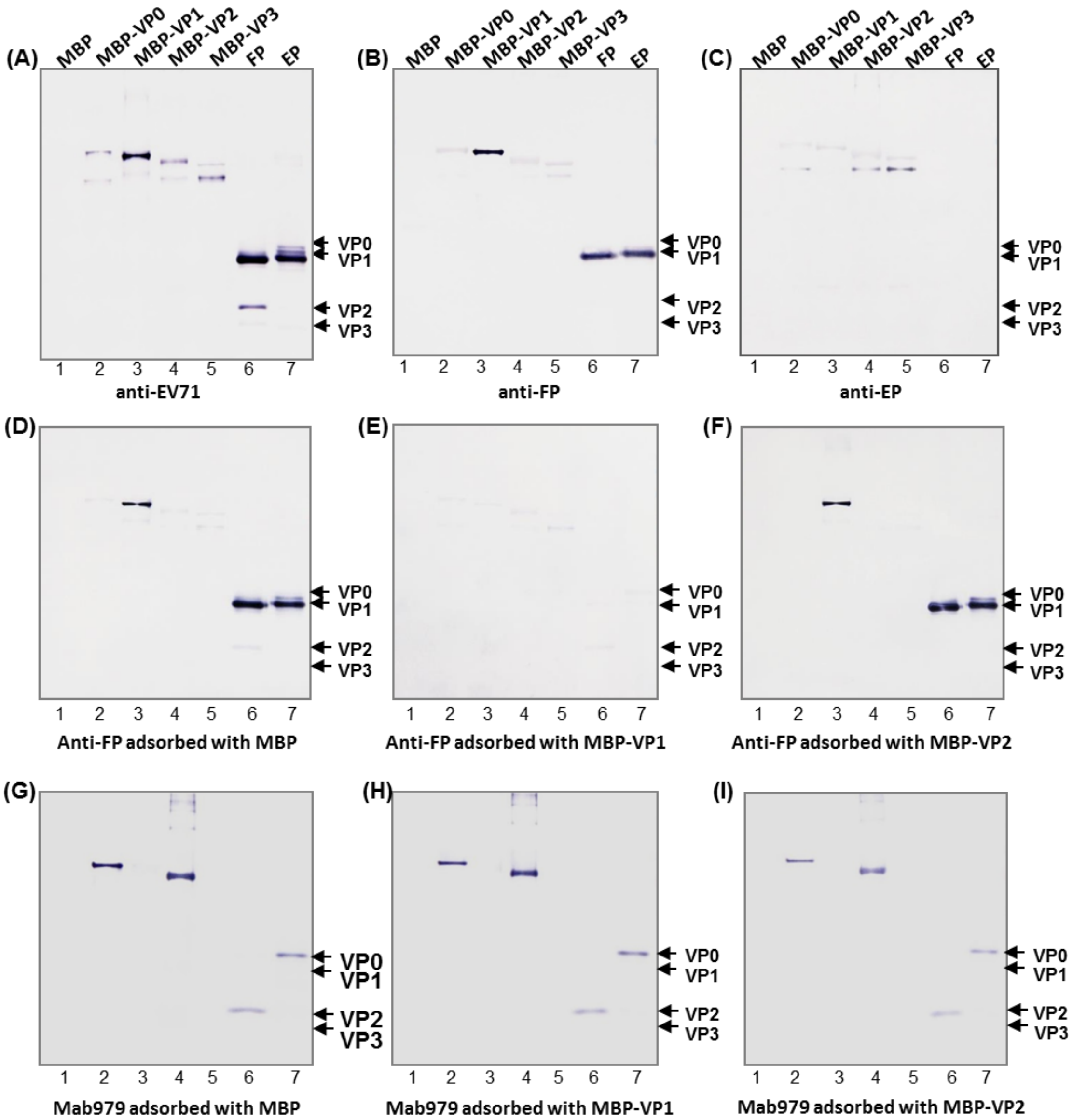

S1 Fig. Western blot analysis of the binding specificity of anti-EV71, anti-FP and anti-EP. Protein samples of MBP, MBP-VP0, MBP-VP1, MBP-VP2, MBP-VP3, FPs and EPs from EV71 were separated on a 10% SDS-PAGE gel and transferred to a PVDF membrane for western blot analysis against the (A) anti-EV71 vaccine, (B) anti-FP, and (C) anti-EP. The binding titer reduction after protein adsorption treatment was confirmed by western blot analysis with probes consisting of anti-FP

adsorbed with MBP (D), anti-FP adsorbed with MBP-VP1 (E), anti-FP adsorbed with MBP-VP2 (F), anti-MAB979 adsorbed with MBP (G), anti-MAB979 adsorbed with MBP-VP1 (H), and anti-MAB979 adsorbed with MBP-VP2 (I).
