# Supplementary material for: The mature EV71 virion induced a broadly cross-neutralizing VP1 antibody against subtypes of the EV71 virus
Source: PLoS One. 2019 Jan 16;14(1):e0210553. doi: 10.1371/journal.pone.0210553 (PMC6334917; doi:10.1371/journal.pone.0210553)
Supplement: S2 Fig — The Vero cells/microcarriers mixture was sampled from each 1-L spinner flask immediately before infection with EV71 virus (a, c, e, g) and before harvest (b, d, f, h) at varying MOIs. (PDF) [file pone.0210553.s003.pdf]

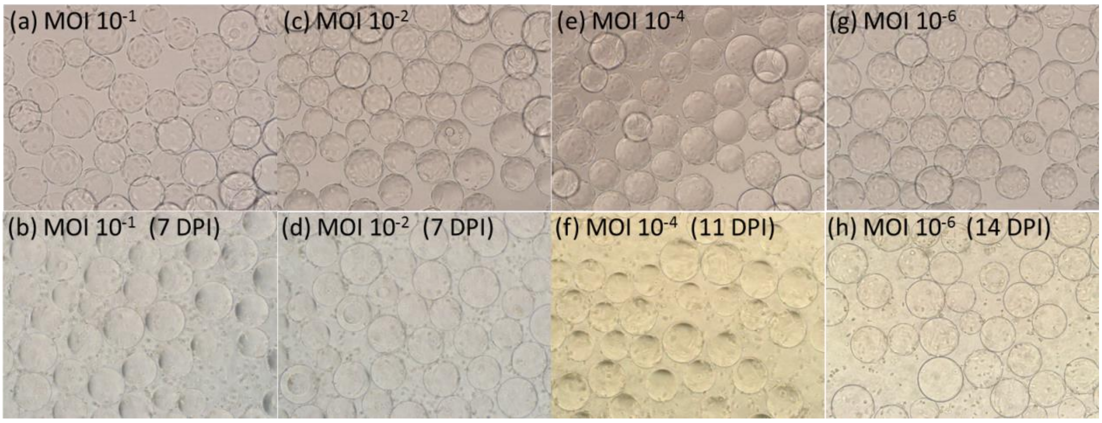

S2 Fig. Photomicrographs of Vero cells on microcarriers. The Vero cells/microcarriers mixture was sampled from each 1-L spinner flask immediately before infection with EV71 virus (a, c, e, g) and before harvest (b, d, f, h) at varying MOIs.
