# Supplementary material for: The mature EV71 virion induced a broadly cross-neutralizing VP1 antibody against subtypes of the EV71 virus
Source: PLoS One. 2019 Jan 16;14(1):e0210553. doi: 10.1371/journal.pone.0210553 (PMC6334917; doi:10.1371/journal.pone.0210553)
Supplement: S3 Fig — Rabbits were immunized with FPs to generate antibodies that recognized FPs or EPs. In the competitive ELISA, the binding specificity for FPs of anti-FP blocked by BSA was defined as 100% (a). The binding specificity for FP of anti-FP blocked by FP was calculated as 0% (b). The binding specificity for FP of anti-FP blocked by EP was calculated as 80% (c). The binding specificity for EP of anti-FP blocked by EP was calculated as 0% (d). (PDF) [file pone.0210553.s004.pdf]

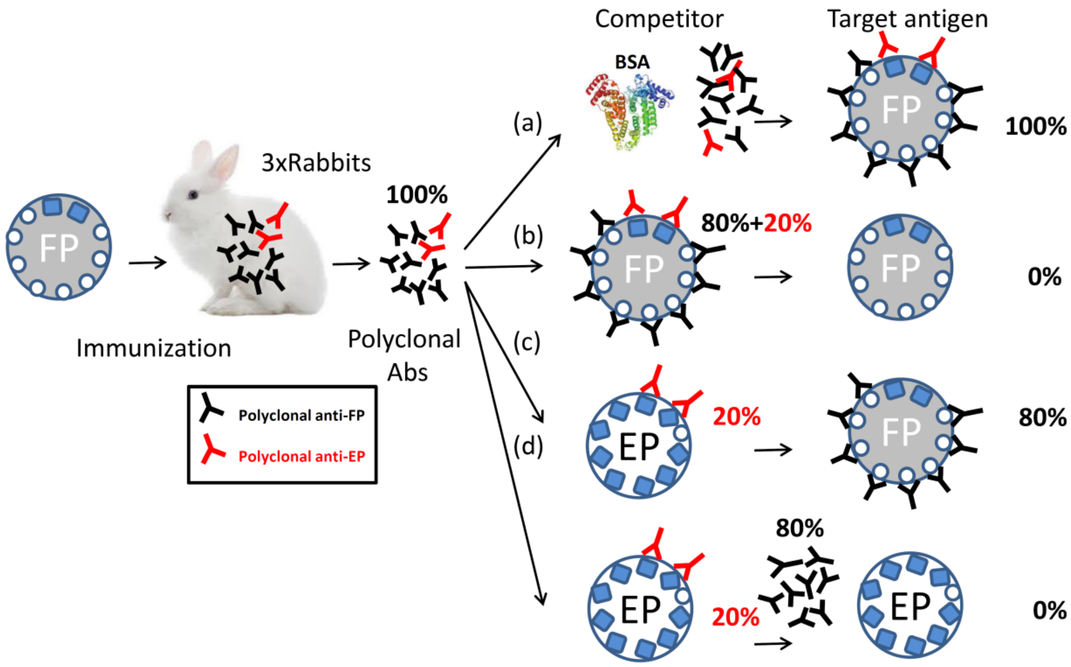

S3 Fig. Proposed mode of specificity for anti-FP binding to FP and EP in the competitive ELISA study. Rabbits were immunized with FPs to generate antibodies that recognized FPs or EPs. In the competitive ELISA, the binding specificity for FPs of anti-FP blocked by BSA was defined as 100% (a). The binding specificity for FP of anti-FP blocked by FP was calculated as 0% (b). The binding specificity for FP of anti-FP blocked by EP was calculated as 80% (c). The binding specificity for EP of anti-FP blocked by EP was calculated as 0% (d).
